# The Nurse Practitioners' Perspective on Inhaler Education in Asthma and Chronic Obstructive Pulmonary Disease

**DOI:** 10.1155/2018/2525319

**Published:** 2018-08-05

**Authors:** Jane Scullion

**Affiliations:** University Hospitals of Leicester NHS Trust, Leicester, UK

## Abstract

Asthma and chronic obstructive pulmonary disease (COPD) can be debilitating conditions adversely affecting a person's quality of life. Effective treatments are available, but common errors in the use of inhalers compound the issue of disease control. The beliefs and concerns of a patient can also have an impact on treatment adherence, the consequences of which are diminished disease control and the occurrence of exacerbations. Once a treatment has been prescribed, it is often nurses who manage the patient long-term, and they may even be the main care provider. This puts nurses in a key position to monitor inhaler technique, communicate with the patient to improve adherence, and even suggest alternative treatments if the patient and therapy are incompatible. This review examines the central role that nurses play in disease management and emphasizes how effective inhaler education can make a difference to disease control. Good communication between the nurse and patient is vital if this is to be achieved. Recent updates to asthma and COPD guidelines are reviewed, and key resources available to help manage patients are highlighted. Finally, with regard to inhaler education, we reconsider the nursing keystones of “Know it,” “Show it,” “Teach it,” and “Review it.”

## 1. Introduction

Asthma and chronic obstructive pulmonary disease (COPD) remain chronic respiratory diseases, with substantial global burden despite currently available treatments and management guidelines [1, 2]. Global prevalence has been estimated at 1–18% for asthma and 12% for COPD, with evidence of increasing incidence over recent years [1, 3]. In 2015 alone, it was estimated that over 3 million people worldwide died from COPD [4]. The availability of effective treatments means that much of this burden is avoidable, and education is pivotal to the implementation of successful intervention strategies [4]. Inhalers are the mainstay of treatment for these diseases [5]. In both disease areas, they improve symptoms and quality of life, and in asthma they save lives. Despite these benefits, there are continued high levels of nonadherence to using inhalers due to both drug and nondrug factors [6].

Nurses are at the forefront of the management of chronic diseases, and in many situations, are the main care provider [7]. In asthma and COPD, both specialist and nonspecialist nurses deliver much of the care that patients receive at primary, secondary, and tertiary levels, playing a key role in the majority of care programs, while in some settings, prescribing nurses also make treatment decisions and changes [8]. As frontline providers of patient care in asthma, nurses are often responsible for the ongoing evaluation of asthma control and for deciding the best treatment in collaboration with the patient and physician [9]. Nurses also play significant roles in most aspects of COPD management, including diagnosis, review and tracking of disease progression and treatment success, and instruction on treatment modification where required [10]. The National Asthma Education Prevention Plan (NAEPP) guidelines advocate that healthcare providers, including nurses, build a strong relationship with their patients through effective communication, answering questions, and supporting effective disease management [9, 11]. These partnerships place nurses in a key position to recognize poor disease control and to provide enhanced care or specialist referral for high-risk patients [9, 11]. Another major aspect of an effective nurse-patient partnership is the opportunity for nurses to deliver patient education, a crucial part of which is inhaler technique [9, 11]. This review will examine, from the nurse practitioners' perspective, the central role that nurses play in the management of asthma and COPD and the essential interventions they can provide to improve patient outcomes, with a particular focus on inhaler education.

## 2. Inhaler Education

Inhalers are the cornerstone treatment for asthma and COPD and are designed to deposit inhaled drug directly to the lungs with minimal systemic side effects [5]. All the most common treatments, such as short- and long-acting *β*_2_-agonists and anticholinergic drugs and corticosteroids, can be delivered in this way. However, the efficacy of treatment and therapeutic outcomes are dependent on a patient's adherence to their dosing regimen and their ability to use their device correctly [12]. The large number of inhalers in the market can be broadly divided into pressurized metered-dose inhalers (pMDI), dry-powder inhalers (DPI), nebulizers, and Soft Mist™ inhalers, each of which is associated with distinct advantages and some common errors in administration technique by the patient (Table 1) [12–15]. Some errors are shared across the majority of prescribed inhalers and typically include preparation, preinhalation expiration, speed and/or depth of inhalation, and postinhalation breath hold [16, 17]. The most common devices to be prescribed for asthma and COPD are pMDIs, but these can be difficult to use due to the high level of coordination required to activate the device while taking a slow and deep inhalation [13, 14, 18]. Indeed, one real-life study involving nearly 3,000 patients recorded that over half of the patients assessed made at least one error when using their inhaler [16]. Use of spacers/valved holding chambers or nebulizers can help overcome some of the coordination difficulties with pMDIs and are routinely used for young children unable to self-manage their inhaler use. However, the practicality and convenience issues of these systems limit their appeal across more patients. Some inhalers are also particularly associated with frequent patient errors. Many patients lack the inspiratory ability to inhale deeply and forcibly, a fundamental requirement of DPI technique that impacts on the particle size that can be generated, which in turn affects drug deposition and therefore efficacy [13]. Patient errors can result in poor disease control and an increase in healthcare utilization [16, 19]. Unfortunately, this high incidence of incorrect inhaler technique has remained at a similar level over the past 40 years despite advances in inhaler development and education [17].

**Figure 1:**
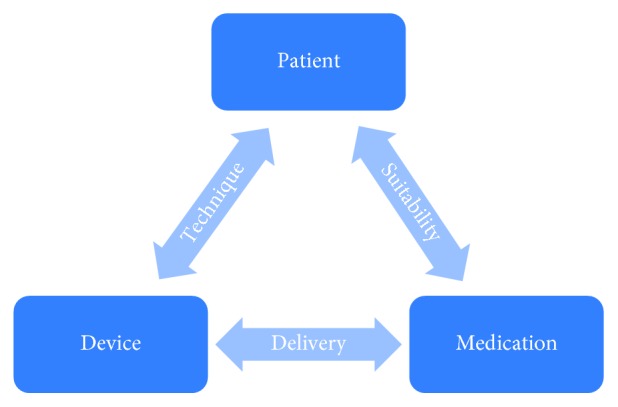
Triangle of patient, medication, and device in chronic obstructive pulmonary disease and asthma.

**Figure 2:**
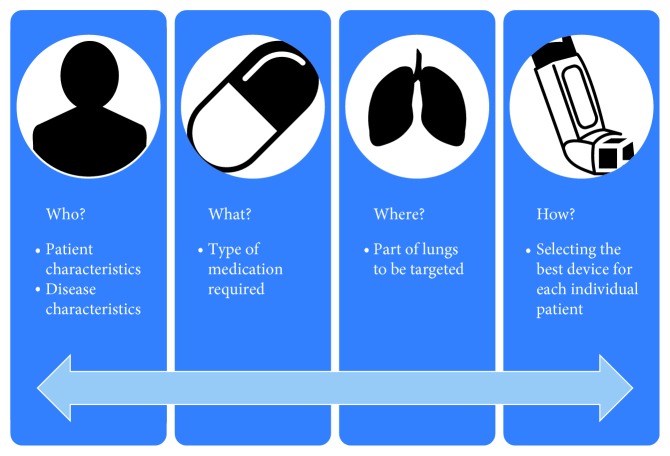
The 3W-H approach for prescribing inhalers [21].

**Figure 3:**
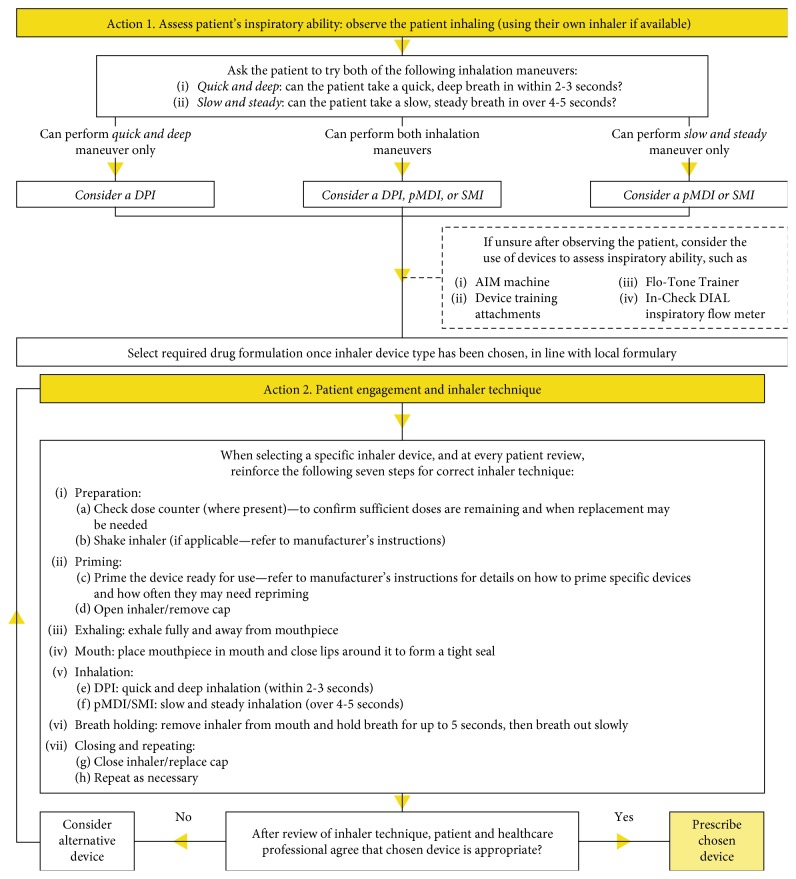
*Guidelines* algorithm for inhaler choice for the treatment of adults with asthma or COPD [22]. COPD: chronic obstructive pulmonary disease; DPI: dry-powder inhaler; pMDI: pressurized metered-dose inhaler; SMI: Soft Mist™ inhaler. Reproduced with permission from Usmani, Capstick, Chowhan, and Scullion. Choosing an appropriate inhaler device for the treatment of adults with asthma or COPD. First published in N. Hayeem, *Guidelines*, vol. 62, pp. 115–117, 2017, MGP Ltd., Chesham, UK, available at http://www.guidelines.co.uk/respiratory/inhaler-choice-guideline/252870.article. This management algorithm was developed by a multidisciplinary expert panel: O. Usmani et al, with the support of a grant from Chiesi Ltd.

For effective management of asthma and COPD, care must be taken in matching each individual patient to the correct medication. The three vital components in this decision—patient, medication, and device—form a triangle, and it is crucial that all three interconnect and complement each other (Figure 1). Each patient should be given optimal medication, be competent in using their device correctly to maximize efficacy, and be happy to use their device [13]. Getting the right inhaler device for people, whether they are children or adults, is not an easy task, and many factors need to be considered, such as age, manual dexterity, cognitive impairment, personal preference, ease of use, inspiratory flow rate, licensing options, and the medication required [20]. Dekhuijzen et al. proposed the 3W-H approach, a very practical method for prescribing inhalers that solely considers the following four simple questions: Who? What? Where? How? (Figure 2) [21]. Patient characteristics to be considered include inspiratory ability and any comorbidities that may affect medication delivery, while achieving the correct diagnosis and evaluating disease severity are also important. Moreover, the medication type and where in the lungs it needs to reach require assessment. The answers to these questions can then be used to match the best device and delivery method to each patient. These questions should be reviewed frequently, as, over time, the answers may change and medication may need to be modified. Recently, an expert panel involving a respiratory consultant, pharmacist, nurse, and general practitioner developed a management algorithm for appropriate inhaler choice in the treatment of adults with asthma or COPD (Figure 3). This dual-action algorithm involves assessment of a patient's inspiratory ability, which can give an indication of a suitable device, and effective patient engagement, including a close observation of inhaler technique [22]. If a patient's medication is changed, it should be remembered that not all inhalers are the same and devices are not interchangeable, so further education will be necessary [23]. Furthermore, the prescribing of multiple inhalers requiring different inhalation techniques should be avoided. A recent study has shown that patients with COPD using several devices with different techniques were more likely to experience exacerbations and be in a higher-dose group than those who used similar technique devices [24]. Similar results have been observed in patients with asthma [25].

The frequent nurse-patient/caregiver interaction at different stages of disease management puts nurses in a prime position to manage patients with asthma or COPD more effectively [9–11]. Using the steps outlined above, nurses have the opportunity to identify patients with poor suitability to a prescribed medication or inhaler. Additionally, nurse-led inhaler education to the patient or their caregiver can positively impact on the crucial “technique” aspect of the decision triangle [9–11]. Nurse-led patient assessment and inhaler education are associated with improved technique, compliance, and patient confidence in asthma, and these effects can be sustained long-term [26]. In a systematic review of randomized controlled trials in asthma, nurse-led educational intervention was demonstrated to significantly improve self-management and self-efficacy [27]. In a study of patients with COPD, nurse-driven patient education was shown to significantly increase inhaler proficiency scores and decrease noncompliant behaviors [28]. Such improvements should have a positive effect on clinical outcomes and may reduce disease morbidity and healthcare utilization.

With inhaler technique identified as the most critical element in asthma and COPD control, it is crucial that those individuals providing the training to the patient are themselves competent in device handling. Healthcare professionals often do not review technique, and even when they do, they are not aware of the correct technique for the device they either prescribe or review [29]. A survey of 150 healthcare professionals reported that 75% provided inhaler training to patients, yet only 7% could demonstrate the correct usage of an inhaler and assessment of inspiratory flow [30]. If healthcare professionals are unaware of, or unable to demonstrate, correct technique, they cannot adequately instruct patients or rectify patient errors.

A patient's inhaler technique should be closely monitored, as bad habits and poor technique can accumulate over time, making it imperative that technique is reviewed and adjusted at every visit if needed [1, 2, 22]. Indeed, the new guidelines “Asthma: diagnosis, monitoring, and chronic asthma management,” issued by the National Institute for Health Care and Excellence (NICE) in the UK, have recommended an assessment of inhaler technique at any asthma review and whenever a new inhaler is prescribed within their “Principles of pharmacological treatment” [31]. The guidelines also recommend the observation of inhaler technique if there is a decline in asthma control, especially following an asthma attack and whenever a patient requests a check [31]. Close monitoring of inhaler technique would also be good practice in COPD care.

## 3. Barriers to Inhaler Adherence and Effective Self-Management

It is important that nurses understand and adapt to the reasons behind a patient's nonadherence to treatment and the key barriers that can impact on inhaler suitability or technique. Nonadherence to therapy may be intentional or unintentional. Unintentional nonadherence may be a result of patient forgetfulness, poor inhaler technique, or inadequate understanding of instructions, all of which could be countered by improved education of patients or their caregivers by nurses and clinicians [32]. Disease- and medication-related beliefs surrounding adverse effects and perceived need for medication can often lead to intentional nonadherence [33]. It is vital that nurses communicate with patients to better understand their motivations, concerns, and preferences [10]. Careful counselling and explanations of the importance of adherence to minimize exacerbations and maximize disease control will be needed to overcome such barriers. Understanding of the patient can be used to tailor treatment appropriately, given that different inhaler characteristics are preferred by different patients [13]. It is important to discuss the ideas, concerns, and expectations (or “ICE”) of the patient or caregiver because if people are not engaged with their healthcare provider, then they are less likely to use their inhaler [34, 35]. Patients are more likely to use their device effectively if they are comfortable with it and can use it, and so they need to be involved in the choice of the device [36].

Physical device characteristics, such as ease of use, convenience, portability, and complexity of instructions, also influence patient satisfaction and adherence [37, 38]. A real-world survey of patients with COPD in Europe found a significant relationship between patient-reported inhaler satisfaction and treatment compliance, and in turn, better compliance was associated with fewer exacerbations and improved quality of life [39]. Similarly, a study in patients with asthma found that inhaler satisfaction was linked with adherence and clinical outcome [40]. Allowing patients to negotiate their preferred therapy or device can impact on treatment adherence. A study by the Better Outcomes of Asthma Treatment (BOAT) group demonstrated that shared decision-making in therapy choices significantly improved adherence to treatment and clinical outcomes [41].

Writing and modification of inhaler prescriptions is restricted to prescribing nurses and clinicians [10], so some nurses will not have the opportunity to assess patient preferences, compatibility, or understanding of a device before it is prescribed. It has been suggested that some patients, because of their natural breathing pattern, may never be able to master the necessary inhalation technique needed for their prescribed inhaler [42]. Attempting to teach a patient on an incompatible device is likely to lead to failure in the long term, emphasizing the need for an individualized approach to match the optimal inhaler type to each patient.

Previous experience of the prescribing healthcare professional can influence device choice and may lead to empirical rather than evidence-based decision-making. This is particularly evident as new and improved inhalers come to market, as healthcare professionals often favor prescribing inhalers with which they are familiar [14]. With an increasing number of devices and choices available, access to up-to-date information will be important to ensure each patient receives optimal care. Guidance on inhaler choice in treatment guidelines is often lacking compared with that for the medication choice itself, and risks prescriptions being based on limited information or presumed cost-effectiveness [43]. For example, recommendations generally do not consider the specific needs of patient subgroups, such as elderly and pediatric populations, or those with low inspiratory flow rates [43]. Nurses are in an excellent position to identify the requirements of these subgroups.

For elderly patients, nurse practitioners can make a big difference. We know that many patients cannot use inhalers correctly, with over 50% of patients struggling to use a pMDI properly. If poor inhaler technique is associated with reduced control and worse COPD outcomes, then an inability to use the inhaler device correctly may account for lack of perceived benefit, which in one study led to 30% of patients with COPD intentionally discontinuing their therapy [44].

Other studies show that between 40 and 60% of patients with COPD do not adhere to their prescribed regimens [45]. In all patients, but especially so with the elderly, adherence can be affected by health beliefs, cognitive ability, and psychological factors. Elderly patients often also have comorbidities that may affect their physical and mental ability to use their device correctly, and societal factors such as access to medications, social support, device training, and follow-up may be pertinent to the elderly [45].

In some countries, prescriptions can be substituted by pharmacists for cheaper, generic versions, which can lead to patients receiving unfamiliar devices. Given the potential differences in inhaler design and required technique between prescribed and substituted inhalers, detrimental effects on patient compliance and clinical outcomes are likely [46]. Although many pharmacists agree with the concerns surrounding this cost-containment practice, few would seek approval on inhaler substitutions from the prescribing healthcare professionals [46]. Nonconsensual switching, that is, not discussed with the patient, can have an impact on patient confidence both in terms of disease control and their relationship with the healthcare profession, which in turn may result in reduced adherence to treatment [47]. To this end, collaborative care between nurse practitioners, physicians, and pharmacists could facilitate improved therapy decision-making and patient outcomes [48].

Failures in self-management support from healthcare professionals can contribute significantly to treatment nonadherence and poor inhaler technique. Lack of time to deliver asthma consultations has been cited by healthcare professionals as a key barrier to achieving effective self-management [49]. There are numerous examples in the literature of insufficient nurse training that could have a direct negative impact on the proficiency of inhaler use by patients. In an evaluation of US inpatient staff nurses' inhaler technique, self-perceived ability was higher than true investigator-measured performance [50]. A questionnaire-based survey of healthcare professionals in the UK found that over 40% of nurses lacked the confidence to construct written action plans for patients, in which inhaler use is fundamental [49]. A similar study revealed that 20% and 52% of UK practice nurses with advanced asthma or COPD roles, respectively, did not have accredited training [51]. Increased support for the healthcare professionals is just as important as patient education.

## 4. Future Considerations

Considering the pivotal role nurses play in effective inhaler education, priority must be given to training nurses in correct device technique and effective demonstration to patients. Successful nurse education is likely to improve outcomes for patients with asthma or COPD and could be delivered through hospital-wide training schemes, one-on-one education, or web-based or unit-based education [50]. However, there is no single standardized educational package for inhaler instruction, unlike the moves to accredit spirometry training in the UK. Standardizing inhaler device training could have an important impact on patient outcomes and is advocated by the UK Inhaler Group (UKIG).

Access to ample placebo devices of different types is important for the education of nurses and patients; however, many nurses do not have access to adequate supplies [15]. Placebo devices can be used by nurses to become familiar with inhalers and to teach the required technique to patients. Patients can also use placebo devices to safely practice and demonstrate their technique [15]. Continuous nurse education is crucial as new medications and devices come to market to ensure patients benefit from these innovative advances in treatment and delivery methods [52].

There are many tools that can support people with the use of their inhaler devices. The In-Check DIAL (Clement Clarke International, UK) can be used to assess the peak inspiratory force a patient can achieve, although it is important to check whether the patient's inspiratory force and technique are consistent with their own device. The Trainhaler (Clement Clarke International, UK) helps to train patients in the correct use of their pMDI by helping them to coordinate actuation of the aerosol during inhalation. There are also training devices such as the Turbohaler Trainer Whistle (AstraZeneca, UK) and the Flo-Tone (Clement Clarke International, UK) that emit a continuous tone if inhalation is correct, which can help encourage patients to use the correct inspiratory flow rate with a pMDI.

Patient or caregiver beliefs and concerns can impact treatment adherence, and they should be factored into nurse training. It can be difficult for a patient with asthma to recognize their need for daily therapy to treat a sporadic illness, especially if they have concerns regarding side effects [32]. Also, healthcare professionals should be aware of religious or cultural beliefs that may impact inhaler use [12]. Nurses should be equipped to reassure patients, put their concerns into context and decide on individual approaches to treatment [10]. Communication skills training is an important area to include in any healthcare education program [11]. There are numerous valuable resources that nurses can utilize to optimize their technique and maximize their knowledge surrounding inhaler choice and competency assessment. An “Inhaler Standards and Competency Document,” published by the UKIG in 2016, contains detailed criteria for inhaler competency assessment, including a general seven-step protocol for inhaler use [53]. Briefly, this outlines the basic steps applicable to all inhalers, the first of which is the proper preparation of both device and dose. Next is the full and gentle exhalation (not into the inhaler) prior to sealing the lips around the mouthpiece of the device. Vitally, the next step is the correct inhalation procedure, which will differ according to the type of device being used (e.g., a slow and steady breath for pMDI or a quick and deep breath for DPI). The final breath hold for up to 10 seconds is critical to ensure the drug reaches the lungs. The last step is the consideration that the process may need to be repeated. Once these factors have been assessed, the specifics for each inhaler device can be addressed and the technique optimized [53]. These seven steps have been reiterated in the recently published management algorithm for the treatment of asthma and COPD (Figure 3) [22].

Asthma UK have various freely available, web-based educational resources intended for patients, including videos demonstrating correct techniques for many types of inhalers [54]. Patients should be encouraged to use these easily accessible resources frequently to maintain good inhaler technique between visits. The rapid rise in digital support available to patients to aid self-management has some benefits [55], but caution is also needed. For example, hundreds of smartphone apps directed at patients with asthma or COPD exist in online stores, but few contain comprehensive information [56–58]. One study found that only one in four of the apps that provided information on inhaler technique for patients with asthma were consistent with current guidelines [57]. It is important for healthcare professionals to know whether patients are using these apps as they may not adhere to current practice. Moreover, there are currently limited data evaluating the impact of these apps on clinical outcomes. Discussions regarding new technology and effective communication between nurse and patient at every visit about all aspects of self-management can ensure a balanced approach using many different resources.

## 5. Conclusions

Inhaler competency is an integral component of effective self-management in asthma and COPD, and nurses play a pivotal role in delivering education to the patient and caregiver that is required to optimize disease control. Nurses are a primary point of contact for patients with asthma or COPD, and so are also in a key position to elicit patients' views on treatments and to encourage treatment adherence. With respect to inhaler technique, it is essential that nurses

- Know it—understand how each device works and learn the techniques required to achieve optimal delivery to the lungs
- Show it—effectively demonstrate to patients how the devices work
- Teach it—teach the correct technique to patients
- Review it—regularly assess technique using inhaler competency criteria, correcting bad habits as required.

To achieve these four principles of nurse-led inhaler education, nurses should be “trained to train” as well as make use of valuable resources such as the inhaler algorithm [22] and competency assessment criteria [53] described in this review.

## Figures and Tables

**Table 1:** Summary of advantages and common errors for inhaler devices [12–15].

Inhaler	Mechanism	Advantages	Common errors
pMDI	Drug suspended/dissolved in propellant	(i) Portable (ii) Multiple metered dose (iii) No contamination of dose (iv) Press and breath requires coordination (v) Quick and easy to use	(i) Inhaler not shaken before use (ii) Inhaler not primed (iii) Incorrect positioning (upright) (iv) Full exhalation prior to dosing (v) Coordination of actuation and inhalation (vi) Poor inhalation technique (vii) Breath hold not long enough

pMDI + spacer	Drug suspended/dissolved in propellant	(i) Less coordination required than for pMDI (ii) Larger doses delivered (iii) Good lung deposition	(i) Inhaler not shaken before use (ii) Inhaler not primed (iii) Incorrect positioning (upright) (iv) Full exhalation prior to dosing (v) Delay between actuation and inhalation (vi) Poor inhalation technique (vii) Breath hold not long enough (viii) Poor maintenance of spacer

BA-MDI	(i) Drug suspended in propellant (ii) Breath-actuated delivery	(i) Portable (ii) Multiple metered dose (iii) No contamination of dose (iv) No coordination required (v) Quick and easy to use	(i) Inhaler not shaken before use (ii) Inhaler not primed (iii) Incorrect positioning (upright) (iv) Full exhalation prior to dosing (v) Poor inhalation technique (vi) Breath hold not long enough

DPI	Breath-actuated delivery	(i) Portable (ii) No coordination required (iii) Quick and easy to use	(i) Incorrect positioning (upright) (ii) Dose not prepared correctly (iii) Full exhalation prior to dosing (iv) Inhalation not forceful enough to actuate dose correctly (v) Breath hold not long enough (vi) Repeated procedures may be needed to administer full dose (vii) Inhaler not stored correctly

SMI	(i) Aqueous solution (ii) Aerosol delivered by compressed spring	(i) Portable (ii) No coordination required (iii) Slow-moving aerosol cloud (iv) Higher lung deposition than pMDI	(i) Full exhalation prior to dosing (ii) Poor inhalation technique (iii) Breath hold not long enough

Nebulizers	(i) Aqueous solution (ii) Aerosol produced by air jet or ultrasonic vibrations	(i) Can be used at any age (ii) Slow-moving aerosol cloud (iii) Tidal breathing	(i) Dose not prepared correctly (ii) Poor maintenance increases risk of bacterial contamination

BA-MDI: breath-actuated metered-dose inhaler; DPI: dry-powder inhaler; pMDI: pressurized metered-dose inhaler; SMI: Soft Mist™ inhaler.
